# Protecting global health partnerships in the era of destructive nationalism

**DOI:** 10.1371/journal.pgph.0004428

**Published:** 2025-04-08

**Authors:** Maya Adam, Desiree LaBeaud, Nokwanele Mbewu, Jennifer Gates, Randall Waechter, Mercy J. Borbor-Cordova, Lydiah Kibe, Sonia Alvarez, Annie Dori, Anil S. Bilimale, Meggie Mwoka, Jessica Deffler, Michele Spring, Avriel R. Diaz, Rachael Farquhar, Willy Dunbar, Comfort R. Phiri, Valerie A. Luzadis, Nicole Redvers, Cristina Alonso, Rosemary Rochford, Rachel Lowe, Kareem Coomansingh, Moses Laman, Rebeca Sultana, Sadie Ryan, Amaya L. Bustinduy, Anita S. Hargrave-Bouagnon, Shazia N. Ruybal-Pesántez, Zebedee Kerry, Trevor Kelebi, Samuel McEwen, Leanne Robinson, Maritza Salazar Campo, Till Bärnighausen

**Affiliations:** 1 Department of Pediatrics and the Center for Digital Health, Stanford School of Medicine, Stanford University, Stanford, California, United States of America; 2 Heidelberg Institute of Global Health (HIGH), Heidelberg University, Heidelberg, Germany; 3 The DG Murray Trust, Cape Town, South Africa; 4 Icahn School of Medicine at Mt. Sinai, Mt. Sinai, New York, United States of America; 5 Caribbean Center for Child Neurodevelopment (CCCN) at Windward Islands Research & Education Foundation (WINDREF), Grenada, West Indies; 6 Faculty of Maritime Engineering and Sea Sciences, Escuela Superior Politécnica del Litoral (ESPOL), Guayaquil, Ecuador; 7 Pacifc International Center for Disaster Risk Reduction (PIC-DRR, ESPOL), Guayaquil, Ecuador; 8 Kenya Medical Research Institute, Eastern Southern Africa Centre of International Parasite Control, Nairobi, Kenya; 9 Papua New Guinea-Australia Transition to Health, Port Moresby, Papua New Guinea; 10 School of Public Health, JSS Medical College, Mysuru, Karnataka, India; 11 Rockefeller Foundation-Boston University 3D Commission, Nairobi, Kenya; 12 Department of Family and Community Medicine, Thomas Jefferson University Hospital, Philadelphia, Pennsylvania, United States of America; 13 Department of Microbiology and Immunology, State University of New York (SUNY) Upstate Medical University, Syracuse, New York, United States of America; 14 Formerly, Armed Forces Research Institute of Medical Sciences (AFRIMS), Bangkok, Thailand; 15 Walking Palms Global Health, Bahía de Caráquez, Manabí, Ecuador; 16 Burnet Institute, Melbourne, Australia; 17 Health Systems and Policies – International Health Research Centre, School of Public Health, Université libre de Bruxelles (ULB), Brussels, Belgium; 18 Zambart, Lusaka, Zambia; 19 State University of New York College of Environmental Science and Forestry and Heart Forward Science, Syracuse, New York, United States of America; 20 Schulich School of Medicine & Dentistry, University of Western Ontario, London, Ontario, Canada; 21 Arctic Indigenous Wellness Foundation, Yellowknife, Northwest Territories, Canada; 22 La Colaborativa, Chelsea, Massachusetts, United States of America; 23 Harvard T.H. Chan School of Public Health, Boston, Massachusetts, United States of America; 24 Department of Immunology and Microbiology, University of Colorado Anschutz Medical Campus, Aurora, Colorado, United States of America; 25 Barcelona Supercomputing Center, Barcelona, Spain; 26 Catalan Institution for Research & Advanced Studies (ICREA), Barcelona, Spain; 27 Centre on Climate Change & Planetary Health and Centre for Mathematical Modelling of Infectious Diseases, London School of Hygiene & Tropical Medicine, London, United Kingdom; 28 Office of Research, Windward Islands Research & Education Foundation (WINDREF), St. George’s University, Grenada, West Indies; 29 PNG Institute of Medical Research, Goroka, Papua New Guinea; 30 International Centre for Diarrhoeal Disease Research, Bangladesh; 31 University of Copenhagen, Copenhagen, Denmark; 32 Institute of Health Economics, University of Dhaka, Dhaka, Bangladesh; 33 Department of Geography and the Emerging Pathogens Institute, University of Florida, Gainesville, Florida, United States of America; 34 Clinical Research Department, London School of Hygiene & Tropical Medicine, London, United Kingdom; 35 Department of Internal Medicine, University of California, San Francisco, San Francisco, California, United States of America; 36 Burnet Institute, Melbourne, Australia; 37 Walter and Eliza Hall Institute, Parkville, Australia; 38 West Sepik Provincial Health Authority, Vanimo, Sandaun, Papua New Guinea; 39 UCI Paul Merage School of Business, University of California Irvine, Irvine, California, United States of America; 40 Africa Health Research Institute (AHRI), Somkhele, KwaZuluNatal, South Africa; PLOS: Public Library of Science, UNITED STATES OF AMERICA

Long-standing global partnerships, critical for protecting the health of human beings and the planet we share, are under attack in 2025. Around the world, a pendulum swing towards nationalism and populism [1] has threatened to destroy international scientific collaborations that took decades to build. Globally, the rise of hard-right extremism jeopardizes fragile structures established to protect the health and human rights of people everywhere [2]. The chaos of haphazard disruption, devoid of accountability, normalizes a lack of perceived responsibility for our fellow human beings [3]. Reckless global socio-political shifts hurt all of us, as citizens of one world, sharing its limited resources and facing common threats of diseases that respect neither borders nor executive orders [4,5].

As scientists and global health advocates, we have dedicated our careers (and much of our lives) to developing and testing innovative solutions that anticipate, prevent, manage and eliminate serious threats to the health of our global community. Our new reality drives us to continue our work. We are accustomed to challenges and recognize their capacity to strengthen our vision for the future. We have learned important lessons, over decades of combined experience and we have joined forces, across the globe, to communicate these broadly [6]. We believe that long-term, trusting, resilient global partnerships have the potential to carry our global community through crises. If the global health community is to weather the current storm, we must rebuild, restore and reinforce our critically important bridges of collaboration, by tethering them to a set of solid, tested foundations [6], summarized here and illustrated in Fig 1.

**Fig 1:**
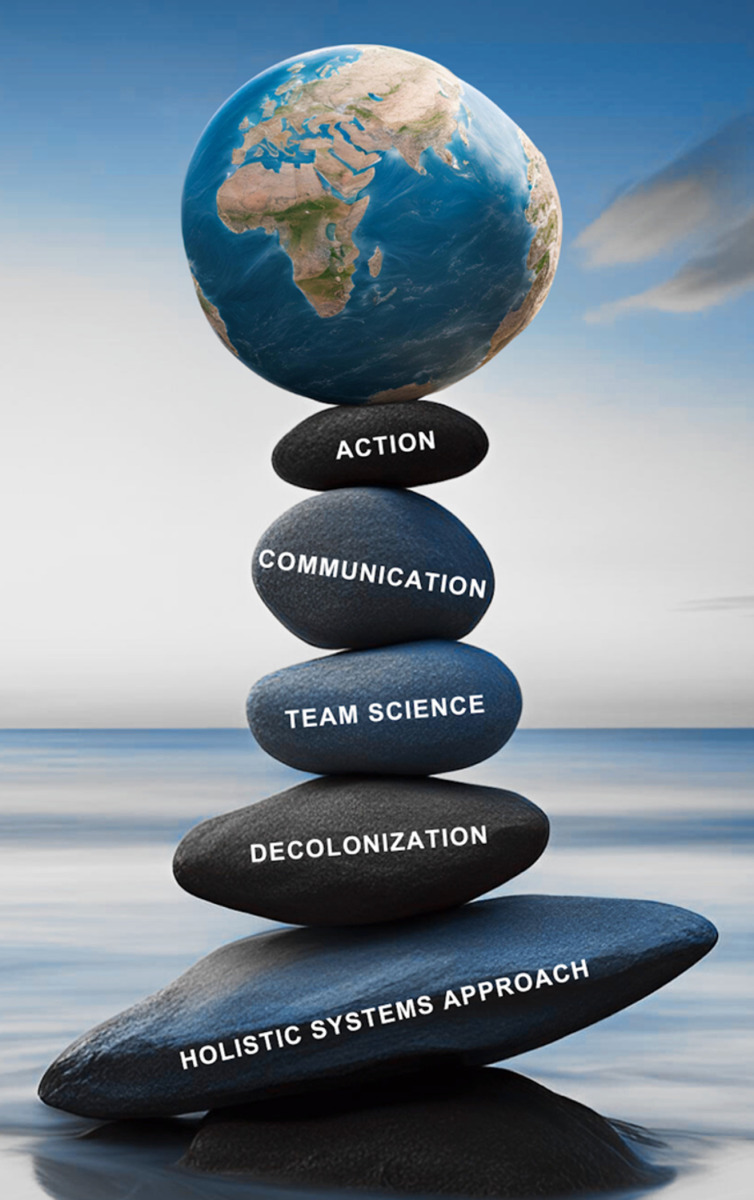
Foundations of global health practice and partnership.

## Holistic systems approach

First, we recognize the interconnectedness and interdependence of the environment, animal, and human systems. The onslaught of recent disease outbreaks have been undeniably linked to climate change. Resulting calls for a unified One Health approach deserve our immediate attention [7]. In the short term, this demands the purposeful creation of a shared agenda, supported by science, in anticipation of a future in which rational governments will prioritize it. To that end, we must persist in hopefulness. Despite current challenges, we have agency, and we can use our partnerships to foster and prepare for needed change.

## Team science is better science

The science underlying a holistic systems approach can only be accomplished through partnerships across scientific disciplines, diverse areas of expertise and lived experience. Team science emerges as a powerful way of conducting scientific research [8]. Most global health challenges are multi-dimensional, so the teams studying them need to include various dimensions of expertise and different perspectives. This involves expanding the boundaries of what is accepted as legitimate science, beyond Euro-western-centric science, to include complementary ways of understanding the world. Collaboration across disciplines involves embracing the diverse, often challenging, roles of global health work and nurturing our different perspectives through adaptive teamwork and flexibility. Furthermore, we must evaluate and iteratively improve our *practice* of team science, using key frameworks, models and approaches to identify procedural strengths and weaknesses. Iterative collaboration goes beyond repetition.

## Decolonizing global health

Throughout history, colonialism has fueled health disparities, disenfranchisement, and high mortality among colonized peoples. When powerful nations impose systems that neglect certain groups, major health disparities are inevitable. We must acknowledge these origins of global health disparities, educate ourselves on the history of the place and the people with whom we partner, and work to address the effects of colonization. For example, local communities are central to the co-creation process, if the resulting solutions are to be effective. We must seek mechanisms for equipping and supporting local leaders, while we apply anti-racism, gender, and intersectional frameworks to redress historical injustices. We must transform funding schemes, educational systems, and research incentives to increase equitable recognition in science, equitable benefits, improved resource sharing, and capacity-building. We need collaborations free of neo-colonial power-hierarchies and structures, characterized by transparency and aligned resource flows. Such global partnerships will benefit the health of marginalized communities worldwide and, in so doing, benefit all.

## Communication matters

Science-based health messages must be delivered in ways that are accessible to diverse, global audiences. This involves meeting people where they are – including where they consume information – and considering their diverse life experiences related to education, language, literacy and cultural affiliation. The design of effective global health communications ideally involves the audiences they aim to reach – creating health messages with them and for them. Strong lines of communication are essential for the promotion of an interconnected and resilient global society. These lines can only be built upon a foundation of mutual respect and shared humanity.

Effective communication among global health partners is equally important and should be centered around trust. Consistently applying ethical principles actively levels power imbalances, promotes gender equity and elevates marginalized voices. We will inevitably work within imperfect systems to drive change. The imperfections challenge all of us to maintain our capacity for empathy and our sense of shared humanity. That sense allows us to experience the joy of basic human connections that powerfully catalyze and sustain our work – and joy is critical for the fulfillment and long-term commitment of individuals and teams. Above all else, if we are to be effective global health partners, we must stay open and humble, practice respectful listening, use authentic storytelling, lay aside biases, self-reflect, learn from our failures, and grow with the process.

## Call to action

Throughout history, human beings have flirted with self-destruction. Globally, the rise of extreme nationalism has fueled xenophobia, racism and withdrawal from international agreements. Populist ideologies have spread unprecedented mistrust in science. Basic human rights have been deprioritized. We have waged brutal wars on one another and on our planet. We have learned, the hard way, that terrible things happen when we fail to work together. As a global team of researchers, physicians, community health advocates, scientists and educators, our work depends on shared leadership as we design human-centered solutions for the health of our world. The shifting socio-political landscape emboldens and further motivates us to realize a different vision for our future – a consensus future characterized by respect, shared humanity and mutual responsibility for our fellow global citizens and the planet we share. Our call to action is both urgent and critical: join us in protecting global health and the partnerships that sustain it.
